# Impact of EMA regulatory label changes on systemic diclofenac initiation, discontinuation, and switching to other pain medicines in Scotland, England, Denmark, and The Netherlands

**DOI:** 10.1002/pds.4955

**Published:** 2020-01-03

**Authors:** Daniel R. Morales, Steve V. Morant, Thomas M. MacDonald, Isla S. Mackenzie, Alexander S. F. Doney, Lyn Mitchell, Marion Bennie, Chris Robertson, Jesper Hallas, Anton Pottegard, Martin Thomsen Ernst, Li Wei, Lizzie Nicholson, Carole Morris, Ron M. C. Herings, Jetty A. Overbeek, Elisabeth Smits, Robert W. V. Flynn

**Affiliations:** ^1^ MEMO Research University of Dundee UK; ^2^ Strathclyde Onstitute of Pharmacy and Biomedical Sciences University of Strathclyde Glasgow UK; ^3^ Dept of Mathematics and Statistics University of Strathclyde Glasgow UK; ^4^ Dept of Clinical Pharmacology and Pharmacy University of Southern Denmark Odense Denmark; ^5^ School of Pharmacy University College London London UK; ^6^ Electronic Data Research and Innovation Service NHS National Services Scotland Edinburgh UK; ^7^ PHARMO Institute for Drug Outcomes Research Utrecht The Netherlands

**Keywords:** diclofenac, impact, pharmacoepidemiology, pharmacovigilance, prescribing, regulation

## Abstract

**Purpose:**

In June 2013 a European Medicines Agency referral procedure concluded that diclofenac was associated with an elevated risk of acute cardiovascular events and contraindications, warnings, and changes to the product information were implemented across the European Union. This study measured the impact of the regulatory action on the prescribing of systemic diclofenac in Denmark, The Netherlands, England, and Scotland.

**Methods:**

Quarterly time series analyses measuring diclofenac prescription initiation, discontinuation and switching to other systemic nonsteroidal anti‐inflammatory (NSAIDs), topical NSAIDs, paracetamol, opioids, and other chronic pain medication in those who discontinued diclofenac. Absolute effects were estimated using interrupted time series regression.

**Results:**

Overall, diclofenac prescription initiations fell during the observation periods of all countries. Compared with Denmark where there appeared to be a more limited effect, the regulatory action was associated with significant immediate reductions in diclofenac initiation in The Netherlands (−0.42%, 95% CI, −0.66% to −0.18%), England (−0.09%, 95% CI, −0.11% to −0.08%), and Scotland (−0.67%, 95% CI, −0.79% to −0.55%); and falling trends in diclofenac initiation in the Netherlands (−0.03%, 95% CI, −0.06% to −0.01% per quarter) and Scotland (−0.04%, 95% CI, −0.05% to −0.02% per quarter). There was no significant impact on diclofenac discontinuation in any country. The regulatory action was associated with modest differences in switching to other pain medicines following diclofenac discontinuation.

**Conclusions:**

The regulatory action was associated with significant reductions in overall diclofenac initiation which varied by country and type of exposure. There was no impact on discontinuation and variable impact on switching.

**KEY POINTS**

- Diclofenac initiation fell following the 2013 EMA regulatory action that varied by country
- EMA regulatory action had a greater effect on diclofenac initiation than discontinuation
- EMA regulatory action was associated with modest differences in switching to other pain medicines
- In Scotland, the EMA regulatory action was associated with an increase in switching to opioids
- Further research is required to better understand variation in the impact of regulatory actions

## 1 INTRODUCTION

Adverse effects from medicines are common, and improving the safe use of medicines requires effective communication of new safety information. Regulatory agencies such as the European Medicines Agency (EMA) and the US Food and Drug Administration (FDA) constantly evaluate the benefit and risk of a medicine and are responsible for alerting prescribers and patients to new safety information. Despite regulatory decisions having the enormous potential to affect public health, their impact is often poorly understood and can vary.^1^

Nonsteroidal anti‐inflammatory drugs (NSAIDs), such as diclofenac, are widely prescribed agents for the management of pain, fever, and inflammatory conditions.^2^ In June 2013, an EMA referral procedure examined the cardiovascular safety of diclofenac based upon evidence from available randomised controlled trials and observational studies.^3^, ^4^, ^5^ The referral procedure concluded that although diclofenac‐containing medicines are effective treatments for their approved indications, systemic formulations of diclofenac were associated with an elevated risk of acute cardiovascular events. The referral procedure also concluded that for the benefit‐risk balance of diclofenac to remain favourable, contraindications, warnings, and changes to the product information, including communication via a direct health care professional communication (DHPC) were required to be implemented across the European Union (EU).

The main elements of the risk minimisation measures were that diclofenac should be used at the lowest dose for the shortest duration possible, and use be contraindicated and cautioned in certain patient groups^6^ In order to evaluate the impact of the risk minimisation measures, the EMA commissioned a study to examine potential changes in various aspects of diclofenac prescribing that may have occurred across several EU countries including a detailed overview of diclofenac initiation, discontinuation, switching to other treatments, and how these were influenced by age, gender, and indication. The aim of this study was therefore to evaluate the impact of the EMA risk minimisation measures implemented in 2013 on overall diclofenac prescription initiation, discontinuation, and switching to other prescribed pain medication in Denmark, The Netherlands, England, and Scotland.

## 2 METHODS

### 2.1 Data sources

Four validated population data sources were analysed (see supplementary methods for details). In brief, these were the following:

- The United Kingdom Clinical Practice Research Datalink (CPRD) that contains primary care data. For this analysis, we used only up‐to‐standard data from non‐Scottish practices (encompassing England, Wales, and Northern Ireland—henceforth, referred to as “England”) with the majority (approximately 90%) being from England.^7^
- The Scottish Prescribing Information System (PIS) that records all medicines dispensed from pharmacies in Scotland that can be record‐linked to demographic data, Scottish Morbidity Records, and death registrations for the entire population.^8^
- The Danish Register of Medicinal Products that records all out‐of‐hospital prescriptions and allows linkage of drug exposures to inpatient and outpatient hospital contacts and death data.^9^, ^10^, ^11^
- The Dutch PHARMO Database Network combines data from primary and secondary health care settings in The Netherlands. These different data sources, including data from general practices, in‐ and out‐patient pharmacies, clinical laboratories, hospitals, the cancer registry, pathology registry and perinatal registry, are linked on a patient level through validated algorithms.^12^

### 2.2 Study population

Cohorts were generated to provide aggregate time series data for analysis using a common protocol (EU PAS Register number EUPAS24089).^13^ The study start period varied by the availability of data from each database. For each country, data were available for 2007Q1 to 2018Q1 in Denmark; 2008Q2 to 2016Q4 in The Netherlands; 2007Q1 to 2018Q1 in England; and 2010Q3 to 2017Q4 in Scotland. All patients were required to have at least 1 year of observation (lookback period) prior to inclusion in the cohort. Cohort entry was defined within each data source by the date of registration with the general practice (in CPRD and PHARMO data sources) or date of first recorded prescription or any secondary care diagnosis (in Danish and Scottish data sources). A patient's index date was the latest of the study period start date, the date of birth, or their first database follow up date plus 1 year (to allow sufficient time to determine prevalent versus incident use of medicines). A patient's last follow up date was the first occurrence of the following: death (all databases); end of study period (which varied between countries); and end of registration (end of registration would not significantly affect data from Denmark and Scotland because they use national data that captures patients moving within the health system). A patient was included in the time period aggregate if the first and last day both lay between the patient's index date and their last follow up date, so patients were observable for the entire quarter.

### 2.3 Exposures

The study population consisted of patients with the exposures listed below (see supplementary methods for full definition). These were analysed as a series of proportions from aggregated patient counts evaluated in each quarter over the study period.

- Diclofenac prescribing initiation overall
- Diclofenac prescribing initiation by indication, age, gender, and exposure type
- Diclofenac prescribing discontinuation overall
- Switching patterns to other prescribed pain medicines following diclofenac discontinuation

Exposure type was defined as one‐off use, sporadic use or chronic use, and we also calculated the mean prescription duration for diclofenac (please see supplementary methods for further details of definitions).

### 2.4 Outcome

The outcome of interest evaluated was any immediate change in prescribing at the date of the regulatory action (pre‐specified as June 2013, 2013Q2) and/or change in prescribing trend postintervention compared with the baseline prescribing trend.

### 2.5 Study design and statistical analysis

The study design was an interrupted time series regression analysis of prescribing trends. The primary analysis used interrupted time series regression to fit quarterly time trends for each country. The effect of the intervention for each country was represented either by a step function or by a continuous linear function modelling the baseline slope before the intervention time point, the change in slope from the baseline trend to the post‐intervention trend, and the immediate change associated with the intervention time point as described by Wagner et al.^14^ Before fitting all regression models, the data were visualised graphically. The range of data points was trimmed to periods immediately before and after June 2013 where trends were approximated to be linear when discontinuities occurred. Trends were modelled using weighted linear regression, the weights being the denominators in each proportion. All models were checked for autocorrelation using the Durbin‐Watson statistic. All analyses were carried out using SAS V9.4. Please see supplementary methods for further details.

### 2.6 Ethical permissions

Permission to conduct the study in each database was obtained from the relevant source from each country, according to each database's standard terms and conditions.

## 3 RESULTS

The overall cohorts consisted of approximately 5.6 million in Denmark, approximately 5.3 million in Scotland, approximately 4.2 million in England, Wales and Northern Ireland, and approximately 1 million in The Netherlands. The quarterly prevalence of diclofenac initiation at the start and end of each data source follow‐up ranged from 0.98% in Denmark to 2.47% in the Netherlands (Table S1).

### 3.1 Impact of the 2013 EMA intervention on diclofenac initiation

**Figure 1:**
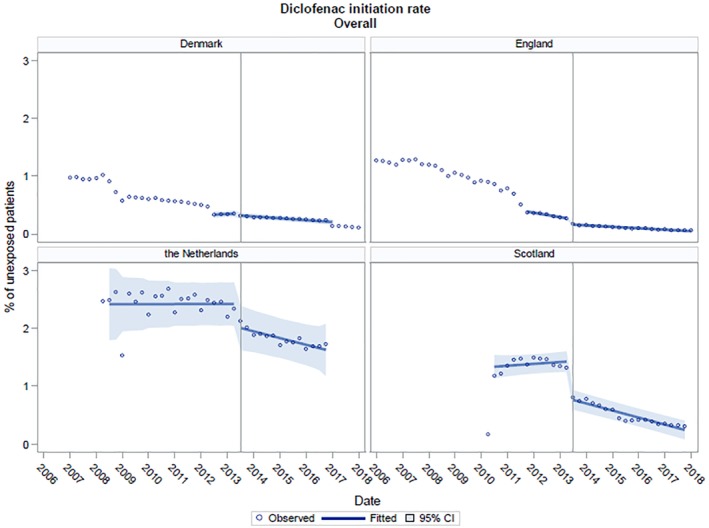
Trends in diclofenac initiation in Denmark, the Netherlands, England, and Scotland

Results of overall diclofenac initiation per country are shown in Figure 1 and Table 1. In Denmark, the Netherlands, and Scotland, there was no trend in diclofenac initiation before the regulatory action, whilst in England, the trend was negative. In England, The Netherlands, and Scotland, the regulatory action was associated with a significant immediate fall in diclofenac initiation (Table 1). Postintervention, diclofenac initiation fell less steeply in England, began to significantly fall in The Netherlands and in Scotland (Table 1). The regulatory action was not associated with a significant change in diclofenac initiation overall in Denmark.

**Table 1:** Interrupted time series regression results for trends in overall diclofenac initiation and discontinuation in each country

	Trends in Diclofenac Initiation and Discontinuation Rates (%/quarter)		
	Before June 2013	Change in first quarter after June 2013	Change after June 2013
Initiation overall			
Denmark	0.006 (−0.014, 0.026), *P* = .534	−0.038 (−0.096, 0.020), *P* = .183	−0.014 (−0.034, 0.006), *P* = .156
England^a^	−0.019 (−0.022, −0.016), *P* = <.001	−0.093 (−0.109, −0.077), *P* = <.001	0.013 (0.009, 0.016), *P* = <.001
Netherlands	0.000 (−0.016, 0.016), *P* = .970	−0.417 (−0.658, −0.175), *P* = .001	−0.029 (−0.057, −0.001), *P* = .041
Scotland	0.008 (−0.006, 0.022), *P* = .240	−0.671 (−0.790,‐0.552), *P* = <.001	−0.039 (−0.054, −0.023), *P* = <.001
Discontinuation overall			
Denmark	−0.033 (−0.171, 0.105), *P* = .633	−0.680 (−4.889, 3.529), *P* = .745	0.251 (−0.221, 0.722), *P* = .288
England^a^	−1.309 (−1.915, −0.704), *P* = <.001	2.186 (−1.438, 5.809), *P* = .224	0.456 (−0.210, 1.122), *P* = .170
Netherlands	−0.334 (−0.499, −0.170), *P* = <.001	0.797 (−1.957, 3.551), *P* = .559	0.108 (−0.233, 0.449), *P* = .522
Scotland	0.403 (0.049, 0.757), *P* = .027	2.413 (−1.077, 5.903), *P* = .167	−0.932 (−1.383, −0.482), *P* = <.001

a Approximately 10% patients were from Northern Ireland and Wales.

### 3.2 Impact on diclofenac initiation stratified by indication, age, gender, and exposure type

Trends in diclofenac initiation per country by indication, age, gender, and exposure type are shown in Figures S1 to S4. The most common indication for diclofenac in all countries was osteoarthritis. Diclofenac initiation was greater in women than men and consisted mostly of one‐off use. In Denmark, the regulatory action was associated with a significant immediate fall in diclofenac initiation in people with osteoarthritis and change to a negative trend in people with osteoarthritis and inflammatory arthropathies compared with baseline (Table S2). In The Netherlands, the regulatory action was associated with a significant immediate fall in people with osteoarthritis and inflammatory arthropathies, and change to a negative trend for all indications compared with baseline (Table S3). In England and Scotland, the regulatory action was associated with a significant immediate fall for all indications (Tables S4 and S5). When considering all countries, the regulatory action tended to have a greater effect among older patients. In Denmark, it was associated with significant immediate falls and change to a negative trend for chronic use only, compared with England where significant changes were observed with one‐off use and chronic use and in the Netherlands and Scotland where it significantly affected all types of use.

### 3.3 Impact of the 2013 EMA intervention on diclofenac discontinuation

**Figure 2:**
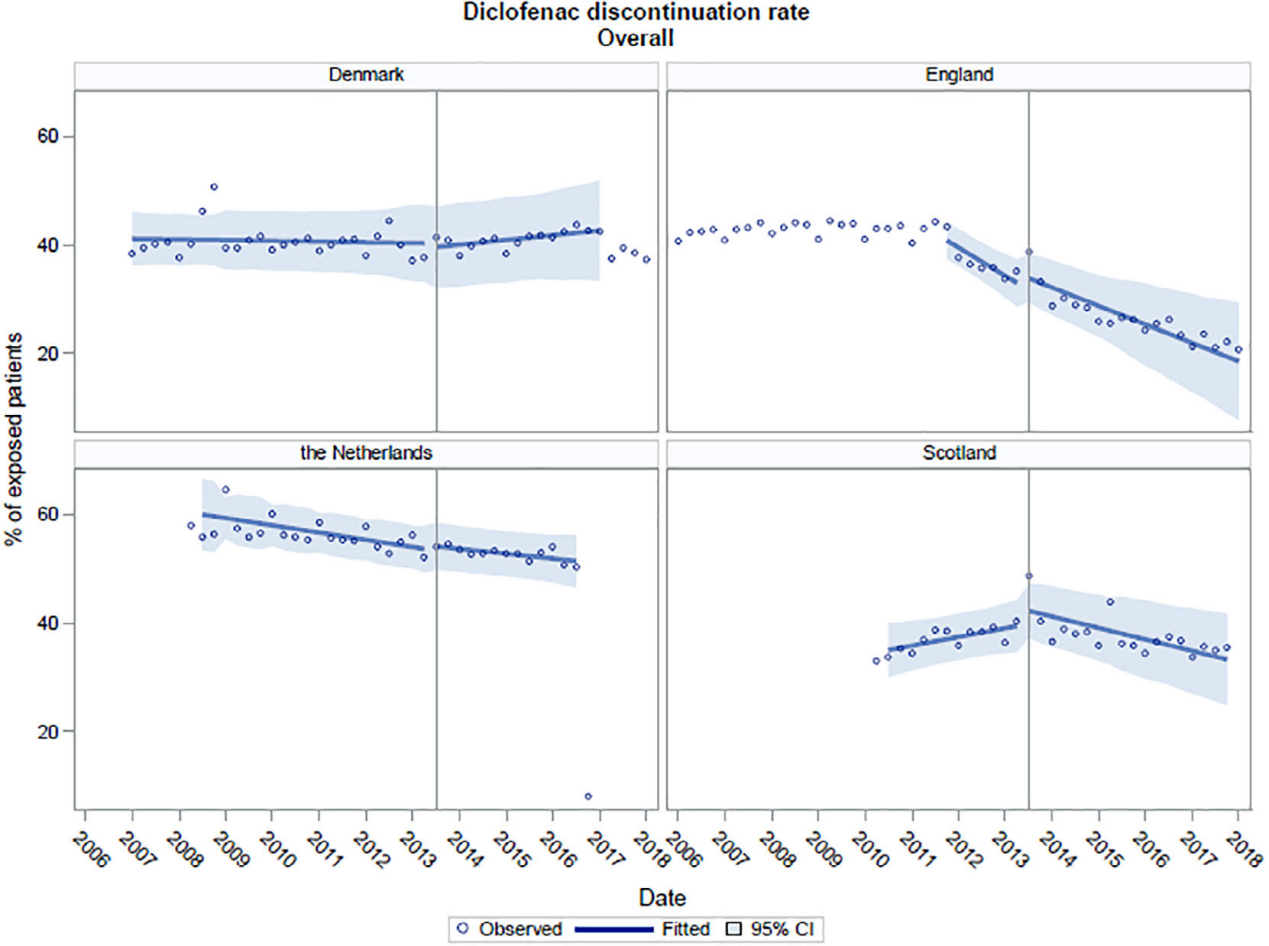
Trends in diclofenac discontinuation in Denmark, the Netherland, England, and Scotland

The results for diclofenac discontinuation per country are shown in Figure 2 and Table 1. The pre‐intervention baseline trend in diclofenac discontinuation was falling in England and The Netherlands, rising in Scotland, and flat in Denmark. The regulatory action was not associated with a significant immediate change in discontinuation or a significant change in trend in Denmark, England, or The Netherlands. In Scotland, the regulatory action was associated with a change from a significantly rising trend to a falling trend in diclofenac discontinuation.

### 3.4 Impact of the 2013 EMA intervention on switching

**Figure 3:**
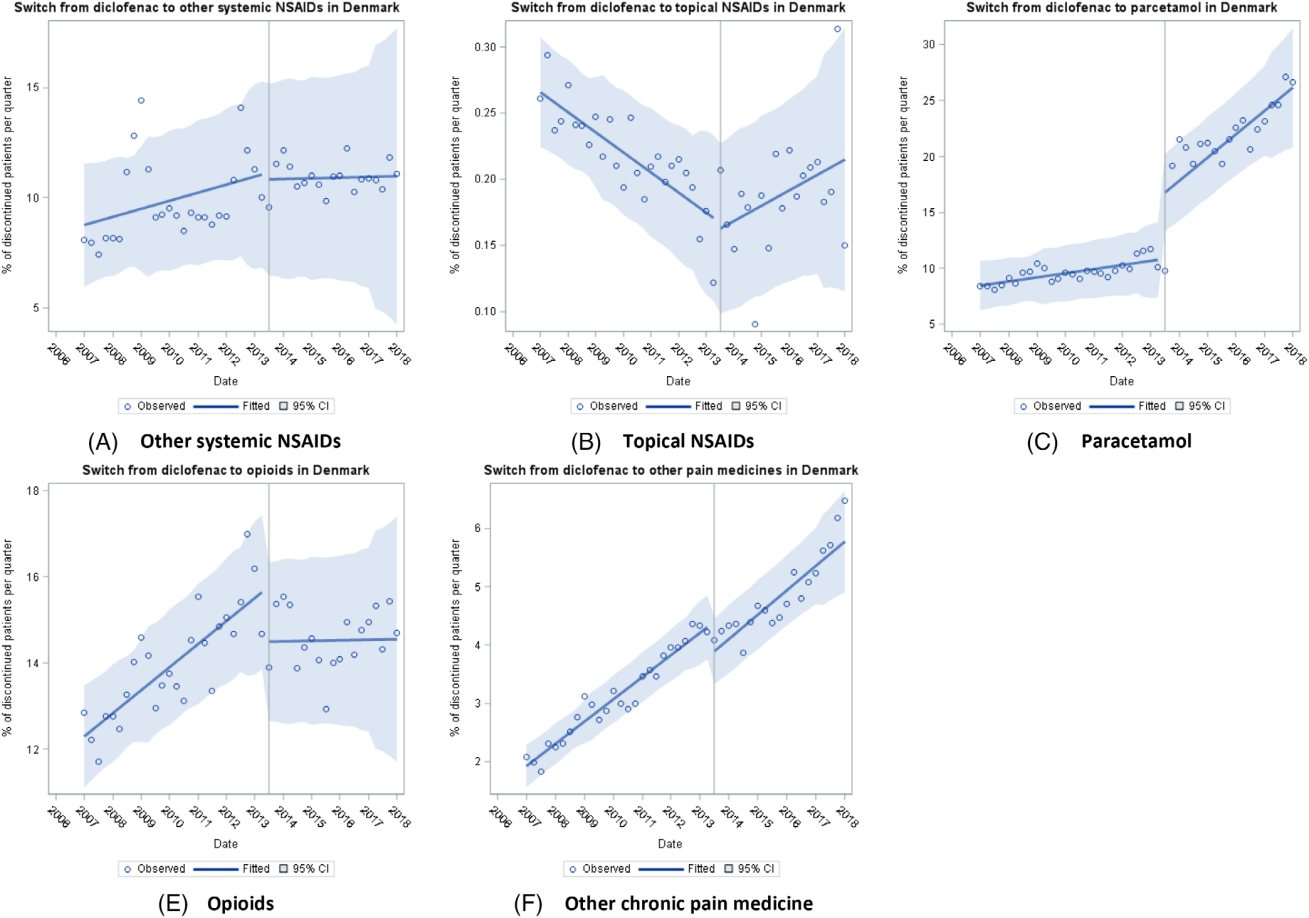
Trends in switching to other pain medicines in Denmark

In Denmark, the baseline trend was rising for switching to other pain medicine groups apart from topical NSAID prescribing that had been falling (Figure 3 and Table 2). The regulatory action was associated with a significant immediate rise in switching to paracetamol (5.92%, 95% CI, 4.07% to 7.77%), and significant immediate fall in switching to opioids (−1.28%, 95% C,I −2.27% to −0.29%) and other chronic pain medication (−0.51%, 95% CI, −0.81% to −0.20%). Compared with baseline, the regulatory action was associated with a significant rising trend in switching to paracetamol and topical NSAIDs and a falling trend in switching to opioids (Table 2). There was no significant change in trend for switching to other systemic NSAIDs or chronic pain medication compared with baseline.

**Table 2:** Interrupted time series regression results for trends in switching to alternative medicines

	Trends in Diclofenac Initiation Rates (%/quarter)		
	Before June 2013	Change in first quarter after June 2013	Change after June 2013
Denmark			
Other systemic NSAIDs	0.091 (0.012, 0.171), *P* = .025	−0.311 (−2.645, 2.022), *P* = .789	−0.083 (−0.301, 0.134), *P* = .443
Topical NSAIDs	−0.004 (−0.005, −0.003), *P* = <.001	−0.004 (−0.039, 0.030), *P* = .803	0.007 (0.003, 0.010), *P* = <.001
Paracetamol	0.092 (0.029, 0.155), *P* = .005	5.920 (4.071, 7.769), *P* = <.001	0.429 (0.257, 0.601), *P* = <.001
Opioids	0.134 (0.100, 0.167), *P* = <.001	−1.279 (−2.266,‐0.293), *P* = .012	−0.131 (−0.222, −0.039), *P* = .006
Other chronic pain medicines	0.095 (0.085, 0.106), *P* = <.001	−0.506 (−0.808,‐0.203), *P* = .002	0.009 (−0.019, 0.037), *P* = .517
The Netherlands			
Other systemic NSAIDs	−0.013 (−0.018, −0.009), *P* = <.001	0.050 (−0.024, 0.124), *P* = .176	‐0.009 (−0.018, 0.000), *P* = .060
Topical NSAIDs	0.002 (0.001, 0.003), *P* = .003	−0.017 (−0.038, 0.005), *P* = .125	0.001 (−0.001, 0.004), *P* = .366
Paracetamol	0.006 (0.000, 0.011), *P* = .043	−0.015 (−0.110, 0.080), *P* = .753	0.001 (−0.010, 0.013), *P* = .849
Opioids	0.015 (−0.033, 0.063), *P* = .525	−0.250 (−1.058, 0.558), *P* = .534	0.052 (−0.047, 0.151), *P* = .290
Other chronic pain medicines	0.008 (0.003, 0.014), *P* = .004	0.037 (−0.054, 0.129), *P* = .413	0.008 (−0.004, 0.019), *P* = .174
England^a^			
Other systemic NSAIDs	0.133 (0.108, 0.159), *P* = <.001	1.509 (0.217, 2.801), *P* = .023	−0.325 (−0.476, −0.174), *P* = <.001
Topical NSAIDs	0.005 (0.002, 0.008), *P* = .001	0.043 (−0.105, 0.191), *P* = .562	−0.014 (−0.031, 0.004), *P* = .121
Paracetamol	0.021 (0.015, 0.026), *P* = <.001	0.251 (−0.036, 0.539), *P* = .085	−0.074 (−0.107, −0.040), *P* = <.001
Opioids	0.020 (0.014, 0.027), *P* = <.001	0.276 (−0.051, 0.603), *P* = .096	−0.065 (−0.103, −0.027), *P* = .001
Other chronic pain medicines	0.046 (0.039, 0.052), *P* = <.001	0.386 (0.048, 0.723), *P* = .026	−0.074 (−0.114, −0.035), *P* = <.001
Scotland			
Other systemic NSAIDs	0.075 (−0.082, 0.233), *P* = .334	5.209 (3.701, 6.716), *P* = <.001	−0.366 (−0.565,‐0.167), *P* = <.001
Topical NSAIDs	0.004 (−0.020, 0.028), *P* = .739	0.352 (0.124, 0.580), *P* = .004	0.025 (−0.005, 0.055), *P* = .099
Paracetamol	−0.019 (−0.043, 0.004), *P* = .106	0.502 (0.277, 0.726), *P* = <.001	−0.053 (−0.083,‐0.024), *P* = <.001
Opioids	−0.009 (−0.018, 0.000), *P* = .058	0.121 (0.036, 0.207), *P* = .007	0.030 (0.018, 0.041), *P* = <.001
Other chronic pain medicines	−0.046 (−0.107, 0.016), *P* = .138	1.305 (0.717, 1.893), *P* = <.001	0.067 (−0.011, 0.144), *P* = .090

a Approximately 10% patients were from Northern Ireland and Wales.

**Figure 4:**
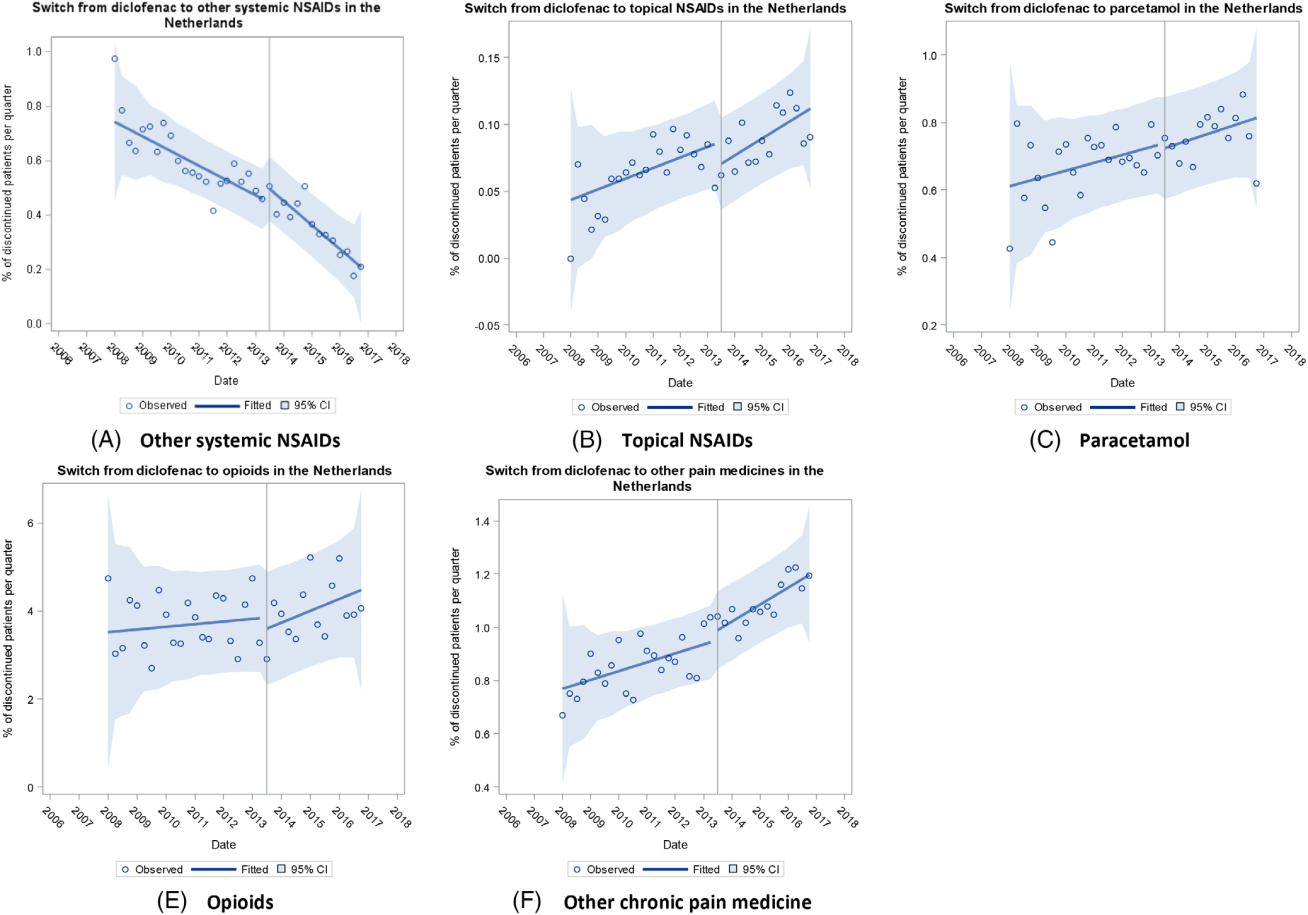
Trends in switching to other pain medicines in the Netherlands

**Figure 5:**
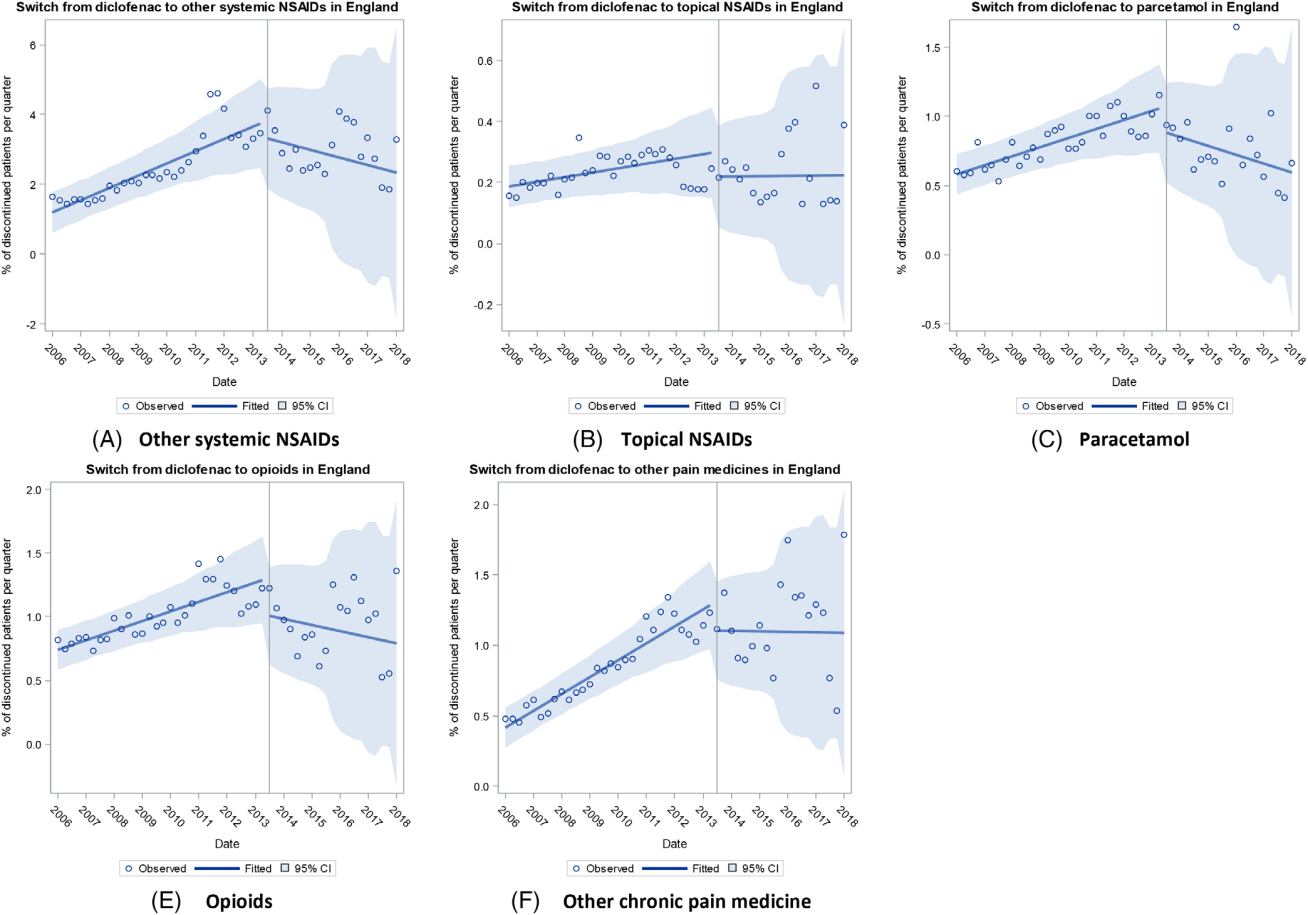
Trends in switching to other pain medicines in England

**Figure 6:**
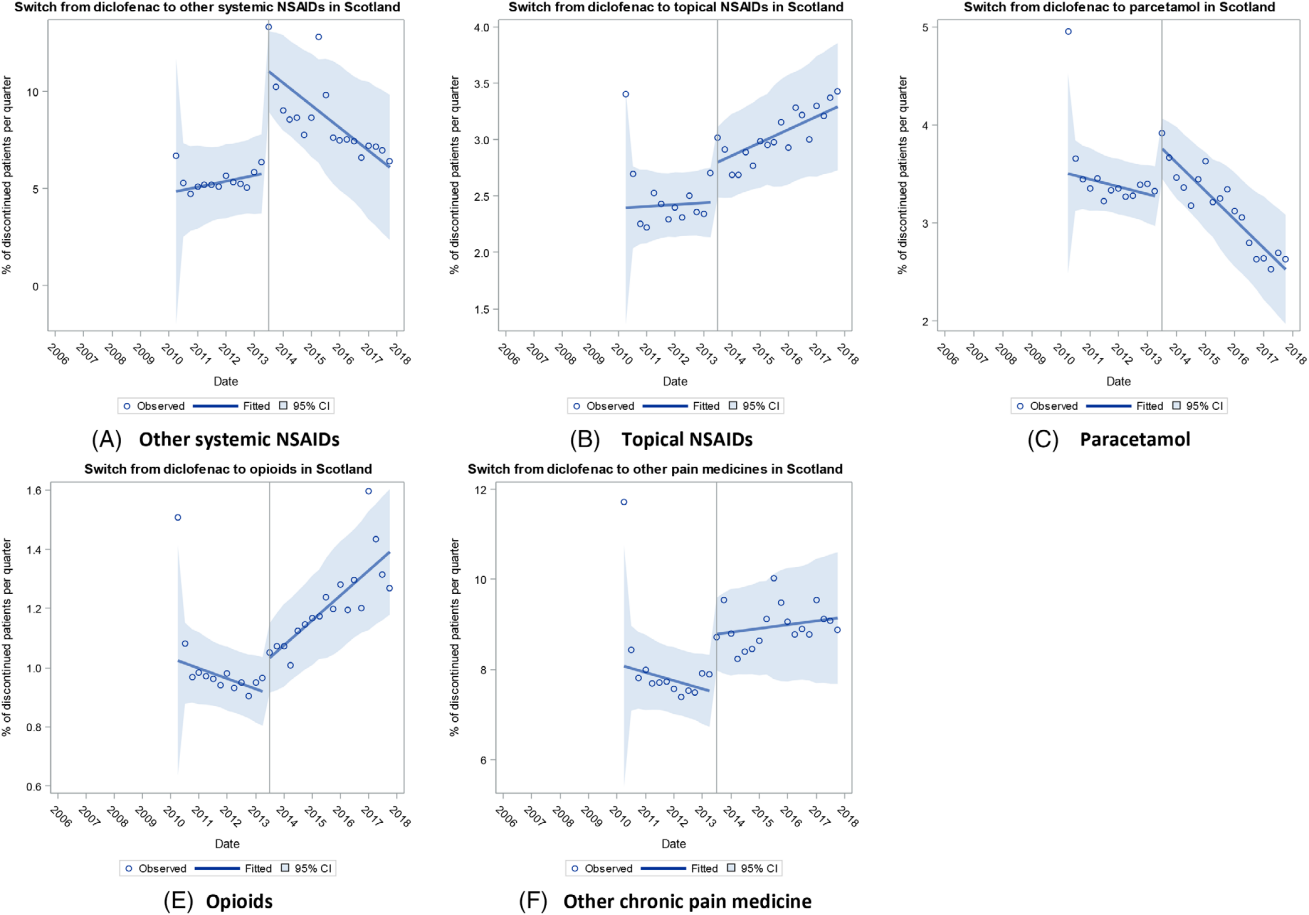
Trends in switching to other pain medicines in Scotland

In The Netherlands, the baseline trend was rising for switching to topical NSAIDs, paracetamol, and other chronic pain medication; falling for other systemic NSAIDs; and flat for opioids (Figure 4 and Table 2). The regulatory action was not associated with any immediate change or change in trend in switching for any group. In England, the baseline trend in switching to other pain medicine groups was rising (Figure 5 and Table 2). The regulatory action was associated with a significant immediate rise in switching to other systemic NSAIDs and other chronic pain medication. Compared with baseline, there was a significant falling trend in switching to all groups following the regulatory action apart for switching to topical NSAIDs that remained unchanged. In Scotland, the baseline trend was flat for switching to other pain medicine groups (Figure 6 and Table 2). The regulatory action was associated with a significant immediate rise in switching to other pain medicine groups and a significantly increasing trend in switching to opioids. In contrast, there were significant falling trends in switching to other systemic NSAIDs and to paracetamol (Table 2).

## 4 DISCUSSION

The 2013 EMA regulatory action for diclofenac had a significant impact on diclofenac prescribing the magnitude and type of which varied between countries. Although the rate of diclofenac initiation fell in all four countries throughout the entire observation period, the 2013 regulatory action for diclofenac was not associated with significant falls in overall diclofenac initiation in Denmark compared with The Netherlands, England, and Scotland. However, it was associated with shortening in the mean duration of diclofenac prescriptions in Denmark and significant impact when examining specific indications for treatment.

Regulators often require a detailed overview on the use of a medicine among the population to assess the whether a risk minimisation measure has been effective. Older adults were most commonly prescribed diclofenac and the impact of the regulatory action tended to be larger in these age groups who are more likely to contain patients with specific contraindications. However, the regulatory action had limited effect on diclofenac discontinuation rates that were highest in The Netherlands (approximately 55%) and lowest in Scotland (approximately 35%). A previous systematic review examining the impact of FDA risk advisories in the United State suggests that such advisories tend to be more effective at decreasing initiation of targeted medicines but less effective at bringing about their discontinuation.^1^ These aspects are important to examine to help understand the key drivers of any change in prescribing, ie, whether health care professionals simply implement the recommendations of the regulatory action in new patients requiring treatment or actively seek to stop existing users. We observed similar differences relating to overall diclofenac initiation and discontinuation within the EU context suggesting these are potentially common responses across heterogeneous health systems. In England and Scotland, diclofenac was often prescribed as a one‐off exposure. This, in combination with a falling initiation rate, may explain the observed fall in diclofenac discontinuation that could be perceived as being counterintuitive.

Given the extent of regulatory actions, published evaluations of their intended or unintended effect on health practitioner behaviour and clinical outcomes are often lacking or subject to methodological limitations.^15^ The net‐benefit of the regulatory actions may be altered if patients are switched to medicines that also cause adverse effects. In this regard, we noted different patterns of switching to other types of pain medicines between countries. In the case of diclofenac and other NSAIDs, one major concern would be a switch to opioids given the opioid epidemic that has received attention globally, and we observed an increase in switching to opioids in Scotland only. However, changes in switching as defined in our study specifically relate to people who discontinue diclofenac only, and the results may not be generalisable to how opioids are prescribed among patients who had not previously used diclofenac.

Limited impact of the 2013 EMA regulatory action for diclofenac has been reported elsewhere in Europe. In a study examining NSAID prescribing data from Lithuania, diclofenac prescribing remained flat over this time and overall NSAID prescribing increased.^16^ However, significant differences should be highlighted. First, compared with Lithuania where diclofenac had the largest NSAID market share, its share in Denmark was as low as 7.3% by volume in 2013.^17^ This may influence the perceived importance of the regulatory action in Denmark and downstream opportunities to publicise information. Second, the baseline trend in diclofenac initiation in Denmark was already falling and continued to fall following the regulatory action. It may be more difficult to directly cause or detect significant changes in prescribing trend if they are already falling (or rising) rapidly in the intended direction, which may similarly have affected the analysis of data from England. Lastly, only a small number of baseline time periods from Denmark were analysed due to obvious changes in trend throughout baseline that would have violated linear assumptions and would have reduced the power to detect statistically significant changes in initiation.^14^, ^18^ These changes in trend within the baseline period may have reflected the earlier safety concerns around diclofenac discussed by the EMA's Committee for Medicinal Products for Human Use (CHMP).^19^

Heterogeneity in impact may also relate to features such as the type of warning, method of dissemination, clinical context, media coverage, changes in guidelines, and public and professional perceptions that risks are serious.^20^ For example, the 2004 UK risk communication on the use of antipsychotics in dementia containing clear recommendations led to greater changes in prescribing in Scotland compared with the 2009 less specific communication in a drug bulletin.^21^ The 2004 risk communication was associated with a switch to typical antipsychotics that were considered safer at the time but were later shown to be associated with similar risks. This suggests that monitoring the impact on switching can be important to detect unintended consequences early.

Key strengths of this study are the use of large, high quality data sources, and a common protocol to standardise data obtained from each data source. This study has several limitations, however. First, our data sources do not capture over the counter use of pain medication. Second, although interrupted time series analysis is a robust quasi‐experimental design to evaluate the effects of policy interventions, it examines associations around a pre‐specified time period and prescribing behaviour may be affected by other factors occurring simultaneously at other points in time.^14^ However, this and other related time series methods are recognised as a strong study design to answer these types of questions, in a field that historically has often used much weaker study designs to attempt to answer similar questions.^15^, ^22^ The duration of follow up available for analysis varied according to each data source resulting in different numbers of data points used within regression analyses.

We demonstrate that variation in impact clearly exists, but it can be challenging to determine whether a regulatory action is considered successful when no established thresholds exist, or perhaps more pertinently, whether further regulatory action would be required to reinforce warnings should be considered. Further information examining what impact the regulatory action had on targeted populations in whom diclofenac was contraindicated or cautioned would support those decisions. The effective implementation of regulatory actions is complex and requires input from multiple stakeholders, but there is some evidence demonstrating which types of dissemination and communication methods are most effective.^20^ It seems imperative that evaluating the impact of regulatory actions should routinely account for both intended and unintended consequences on either prescribing health outcomes or both. In conclusion, the EMA 2013 regulatory action targeting systemic diclofenac products reduced overall diclofenac initiation the extent of which varied by country, whilst having limited impact on diclofenac discontinuation and variable impact on switching to other pain medicines.

## CONFLICT OF INTEREST

E.S., J.O., and R.H. are employees of the PHARMO Institute for Drug Outcomes Research. This independent research institute performs financially supported studies for government and related health care authorities and several pharmaceutical companies.

T.M.M.'s university holds research grants from Novartis, Ipsen, Teijin & Menarini. He is or has been the Principal Investigator on trials paid for by Novartis, Ipsen, Teijin, RTI, GlaxoSmithKline, SHIRE and Menarini. In the last 3 years, he has been paid consulting fees by Novartis and Merck. None of these studies relate to diclofenac.

## AUTHOR CONTRIBUTIONS

All authors were involved in the study design, approval of the study protocol, interpretation of results, and drafting of the manuscript.

## Supporting information


**Data S1.** Supplementary Methods
**Figure S1.** Trends in diclofenac initiation in Denmark, the Netherlands, England and Scotland by indication.
**Figure S2.** Trends in diclofenac initiation in Denmark, the Netherlands, England and Scotland by age.
**Figure S3.** Trends in diclofenac initiation in Denmark, the Netherlands, England and Scotland by gender.
**Figure S4.** Trends in diclofenac initiation in Denmark, the Netherlands, England and Scotland by exposure type.
**Figure S5.** Trends in the mean duration of diclofenac prescriptions in Denmark, the Netherlands, England and Scotland in days.
**Table S1.** Prevalence of diclofenac initiation at the beginning and end of follow‐up for each country.
**Table S2.** Interrupted time series regression results for trends in diclofenac initiation in Denmark by indication, age, gender, exposure type and prescription duration.
**Table S3.** Interrupted time series regression results for trends in diclofenac initiation in the Netherlands by indication, age, gender, exposure type and prescription duration.
**Table S4.** Interrupted time series regression results for trends in diclofenac initiation in the England by indication, age, gender, exposure type and prescription duration.
**Table S5.** Interrupted time series regression results for trends in diclofenac initiation in the Scotland by indication, age, gender, exposure type and prescription duration.
**Table S6.** List of medicines used in the study.
