# Supplementary material for: Impact of EMA regulatory label changes on systemic diclofenac initiation, discontinuation, and switching to other pain medicines in Scotland, England, Denmark, and The Netherlands
Source: Pharmacoepidemiol Drug Saf. 2020 Jan 3;29(3):296–305. doi: 10.1002/pds.4955 (PMC7079064; doi:10.1002/pds.4955)
Supplement: Supplementary file 1 — Data S1. Supplementary Methods Figure S1. Trends in diclofenac initiation in Denmark, the Netherlands, England and Scotland by indication. Figure S2. Trends in diclofenac initiation in Denmark, the Netherlands, England and Scotland by age. Figure S3. Trends in diclofenac initiation in Denmark, the Netherlands, England and Scotland by gender. Figure S4. Trends in diclofenac initiation in Denmark, the Netherlands, England and Scotland by exposure type. Figure S5. Trends in the mean duration of diclofenac prescriptions in Denmark, the Netherlands, England and Scotland in days. Table S1. Prevalence of diclofenac initiation at the beginning and end of follow‐up for each country. Table S2. Interrupted time series regression results for trends in diclofenac initiation in Denmark by indication, age, gender, exposure type and prescription duration. Table S3. Interrupted time series regression results for trends in diclofenac initiation in the Netherlands by indication, age, gender, exposure type and prescription duration. Table S4. Interrupted time series regression results for trends in diclofenac initiation in the England by indication, age, gender, exposure type and prescription duration. Table S5. Interrupted time series regression results for trends in diclofenac initiation in the Scotland by indication, age, gender, exposure type and prescription duration. Table S6. List of medicines used in the study. [file PDS-29-296-s001.pdf]

Impact of EMA regulatory label changes on systemic diclofenac initiation, discontinuation and switching to other pain medicines in Scotland, England, Denmark and The Netherlands

SUPPLEMENTARY CONTENT

Daniel R Morales[1]
Steve V Morant[1]
Thomas M MacDonald[1]
Isla S Mackenzie[1]
Alexander S F Doney[1]
Lyn Mitchell[1]
Marion Bennie[2]
Chris Robertson[2]
Jesper Hallas[3]
Anton Pottegard[3]
Martin Thomsen Ernst[3]
Li Wei[4]
Lizzie Nicholson[5]
Carole Morris[5]
Ron Herings[6]
Jetty A Overbeek[6]
Elisabeth Smits[6]
Robert Flynn[1]

Authors institutional affiliations:

[1] MEMO Research, University of Dundee, UK

[2] University of Strathclyde, UK

[3] University of Southern Denmark, Denmark

[4] University College London, UK

[5]NHS National Services Scotland, UK

[6] PHARMO Institute for Drug Outcomes Research, The Netherlands

SUPPLEMENTARY METHODS

Data sources

CPRD contains data originating from the computer systems of General Practitioners (GPs) across the United Kingdom with an estimated 6 million population, but we used only practices outside Scotland, so data were used from England, Wales and Northern Ireland: for the purposes of this report, these will be referred to as England. The data has been collected since 1987, covers about 7% of the UK population and is broadly generalisable to the whole UK population. For this study, data was be taken from all non-Scottish “up to standard” practices. Data on diagnostic coding and prescribing come from the GP system and are recorded as Read, Gemscript and BNF codes.

The Prescribing Information System (PIS) records all medicines dispensed from pharmacies in Scotland (population estimated 5.3 million in June 2014) and these can be record-linked using the person-unique Community Health Index (CHI) number to demographic data (e.g. age, sex, social deprivation, dates registered with family doctor), Scottish Morbidity Records (e.g. SMR01 – in-patient hospitalisations) and National Records of Scotland (NRS) death registrations for the entire population (International Classification of Diseases (ICD)-9/ICD-10 coded). Prescription data is available from 2009.

The Danish Register of Medicinal Products records all out-of-hospital prescriptions (full population coverage of 5.6 million individuals). A unique 10-digit personal identifier, the Centrale Person register (CPR) number, readily allows linkage of drug exposures to outcomes in the form of ICD-10 diagnoses registered in connection with inpatient and outpatient hospital contacts. Death data is available from the Civil Registration System. Prescription data is available from 1995.

The PHARMO Database Network is a population-based network of electronic healthcare databases and combines data from different primary and secondary healthcare settings in the Netherlands. To address the objectives of the present study the Out-patient Pharmacy and the GP Database were used. The Out-patient Pharmacy Database of the PHARMO Database Network comprises GP or specialist prescribed healthcare products dispensed by the out-patient pharmacy (population 4.2 million in 2016). These data can be linked on a patient-level using probabilistic linkage to other databases. Data on indication and contraindications were obtained from the GP Database for a population of approximately 1 million. This database comprises data from electronic patient records registered by GPs. Dispensing data is recorded as ATC and diagnoses as ICPC (International Classification of Primary Care) codes or entered as free text. ICPC codes can be mapped to ICD codes. Data linked with the GP Database was available from 2007 up to 2016.

Exposures

We used prescription data to calculated non-overlapping periods of exposure and non-exposure to diclofenac over a patients entire observation period. If a prescription occurred before a previous prescription’s end date, the end date of the previous prescription was moved to the day before the current prescription’s start date so that the exposure periods did not overlap. The exposure file was used to create a file of exposure episodes by merging exposure periods separated by less than 92 days of non-exposure and splitting periods of non-exposure into the first 92 days (labelled recently discontinued) and the rest (unexposed). Using 92 days or longer to define discontinuation guaranteed that no patient could discontinue and re-initiate diclofenac in the same quarter.

Diclofenac initiation

Diclofenac initiation was defined as a prescription for diclofenac with no exposure to diclofenac in the preceding 92 days. The denominator was the number of non-users on the first day of the time period defined as no exposure to diclofenac in the previous 92 days. The numerator was the number of these patients initiating diclofenac in the time period. This was performed for overall diclofenac initiation then stratified by indication, age category and gender. Age was classified as 0-17, 18-29, 30-39, 40-49, 50-59, 60-69, 70-79 or 80+.

Read, ICD or ICPC codes were used to classify licenced indications: Crystal arthropathies; Inflammatory arthropathies including Pain and inflammation in musculoskeletal disorders, and Pain and rheumatic disease, including juvenile idiopathic arthritis which were subdivided into osteoarthritis and other inflammatory arthropathies; and osteoarthritis. The classification was based on any record dated before the end of the time point.

One-off users were defined as patients prescribed a single diclofenac prescription only. To define sporadic and chronic users we calculated a possession ratio for each patient defined by using the number of days prescribed (or supplied) assuming a standard daily dose divided by the number of days between diclofenac prescriptions. We defined sporadic users as patients with a diclofenac possession ratio of less than 1 standard day of therapy per 3 days. Patients with a diclofenac possession ratio of more than 1 standard day of therapy per 3 days were defined as chronic users. Standard daily doses were assumed to be:

**Formulation**	**Standard daily dose (mg)**			
	**adults**	**≤ 4 years**	**5-11 years**	**11-18 years**
Tablets or capsules for oral administration	150	50	100	150
Suppositories for rectal administration	150	50	100	150
Solutions for intravenous or intramuscular injection	150	50	100	150

For estimating the duration of each diclofenac prescription, we assumed a standard diclofenac treatment regimen for each patient and prescription as if they were taking it with complete adherence. For tablets/capsules we used a total daily dose 150mg diclofenac. For example, a standard prescription consisting of 50mg strength tablets/capsules we therefore divided the quantity of tablets/capsules per prescription by this standard regimen (i.e. 3) to provide the standard duration of therapy in days. We measured trends in the average standard duration of therapy for prescriptions issued within each time period, before and after the date of the regulatory intervention.

Diclofenac discontinuation

Overall diclofenac discontinuation was defined as the number of patients with a prescription for diclofenac with no exposure to diclofenac in the 92 days following the date of that diclofenac prescription. The denominator was the number of patients prescribed diclofenac in the time period. The numerator was the number of these patients discontinuing.

Switching to other alternative medicines

A switch to an alternative medicine group was defined as those patients who discontinued diclofenac (as defined above) and who then initiated a drug in the class listed in supplementary table S5. Initiation of an alternative medicine was defined as the first prescription of a drug in that class prescribed within 92 days following the date of the last diclofenac prescription.

Date of the regulatory intervention

For interrupted time series regression analysis, the date of the regulatory intervention was pre-specified as 28 June 2013.

Analytical approach

The primary analysis used quarterly time periods. For each year these were defined by the following dates:

- 1st January to 31st March = Quarter 1
- 1st April to 30th June = Quarter 2
- 1st July to 30th September = Quarter 3
- 1st October to 31st December = Quarter 4

The proposed primary analysis used interrupted time series regression to fit time trends to each series of time period data for each country. Using regression modelling we evaluated:

1. The baseline slope before the intervention time point
2. The change in slope from the baseline trend to the post-intervention trend
3. The immediate change associated with the intervention time point

Before fitting all regression models, the data was visualised graphically. The effect of the intervention for each country was represented either by a step function, or by a continuous linear function representing gradual implementation (interrupted time series analysis). This choice, and whether it is necessary to model any trends prior to the intervention time point, was decided on visual inspection of the data. The analysis was done and is reported by data source.

To measure change in trends over time after the pre-specified date of the regulatory intervention, June 2013, this was achieved by fitting interrupted time series (ITS) models with a joint point at this date.[14] They were parameterised so that one parameter estimated the change in slope after vs before the join point (the coefficient of a time variable counting the number of quarters since the intervention and set to 0 before it), and another estimated any step change at the join point (the coefficient of a variable set to 1 after the intervention and 0 before it). Where there was evidence of discontinuities at other times, in either absolute rates or their slopes, the range of data was trimmed to periods immediately before and after June 2013 when trends were approximated to be linear.

We therefore assumed normal error distributions and fitted trends using weighted linear regression, the weights being the denominators in each proportion. We found no increase in the magnitude of residuals with increasing fitted values and therefore did not transform the data to log units for analysis. We performed sensitivity analyses comparing models that did and did not allow for autocorrelation and found trivial differences in the parameter estimates. The analyses presented in this report did not allow for autocorrelation.

SUPPLEMENTARY FIGURES

Figure S1. Trends in diclofenac initiation in Denmark, the Netherlands, England and Scotland by indication.

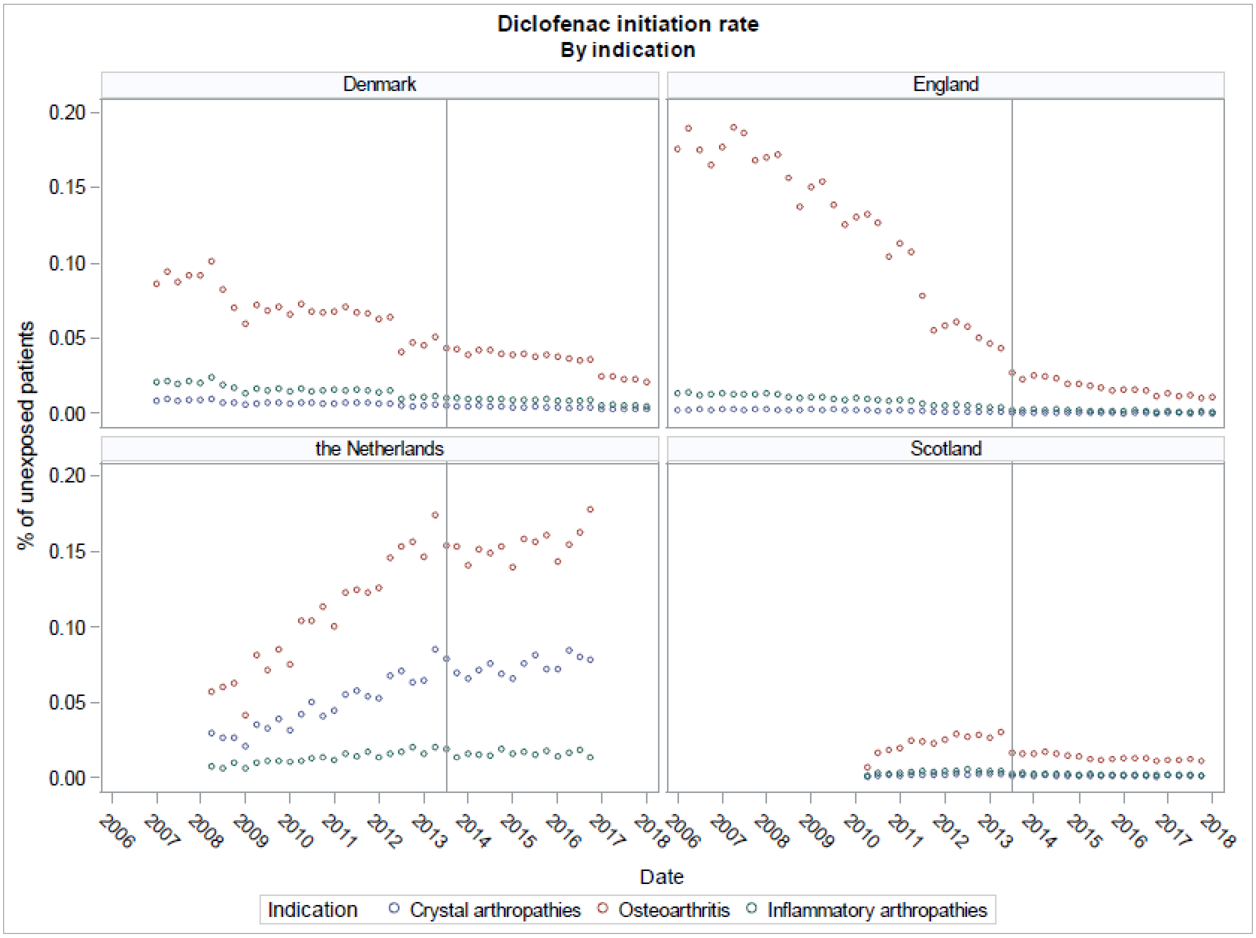

Figure S2. Trends in diclofenac initiation in Denmark, the Netherlands, England and Scotland by age.

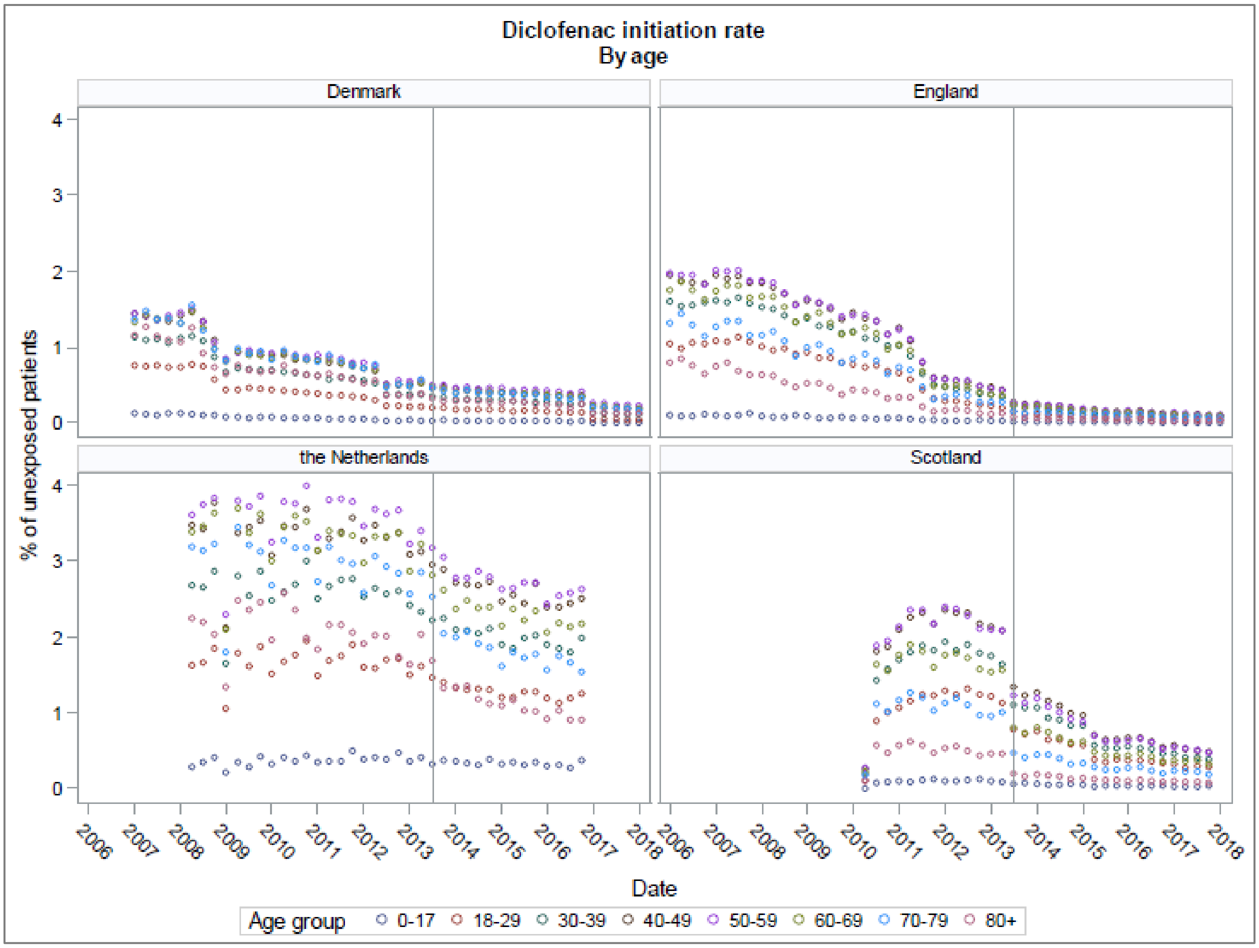

Figure S3. Trends in diclofenac initiation in Denmark, the Netherlands, England and Scotland by gender.

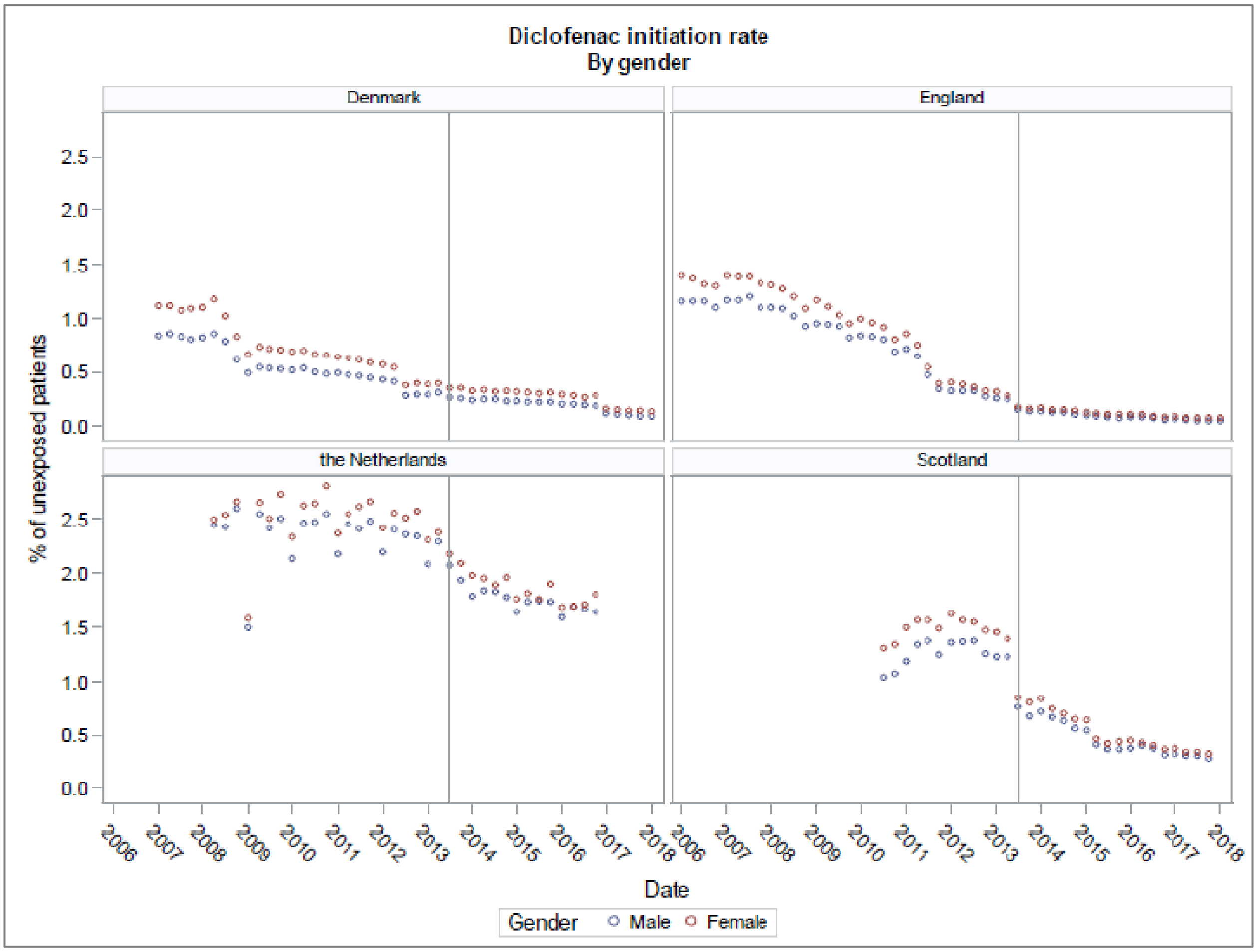

Figure S4. Trends in diclofenac initiation in Denmark, the Netherlands, England and Scotland by exposure type.

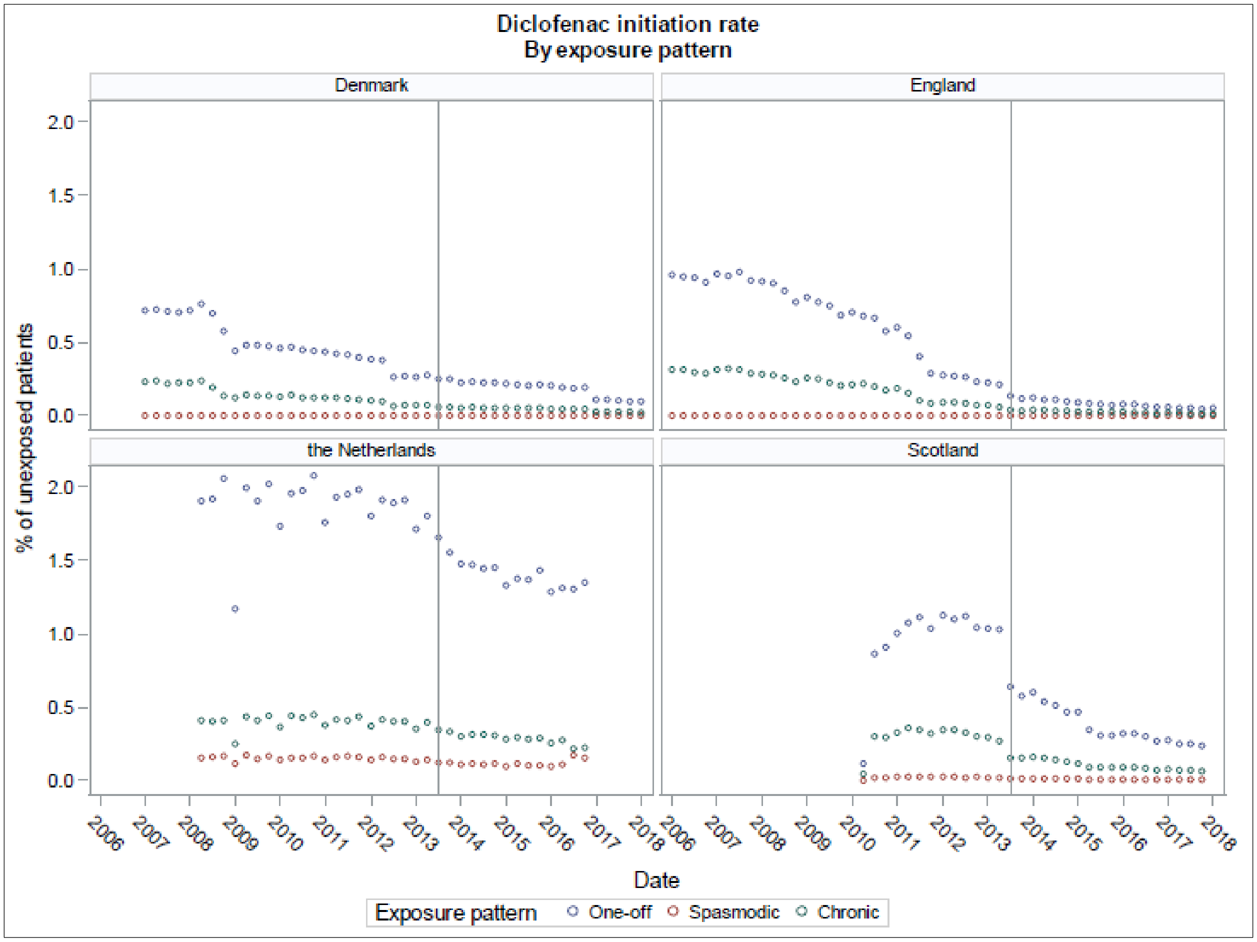

Figure S5. Trends in the mean duration of diclofenac prescriptions in Denmark, the Netherlands, England and Scotland in days.

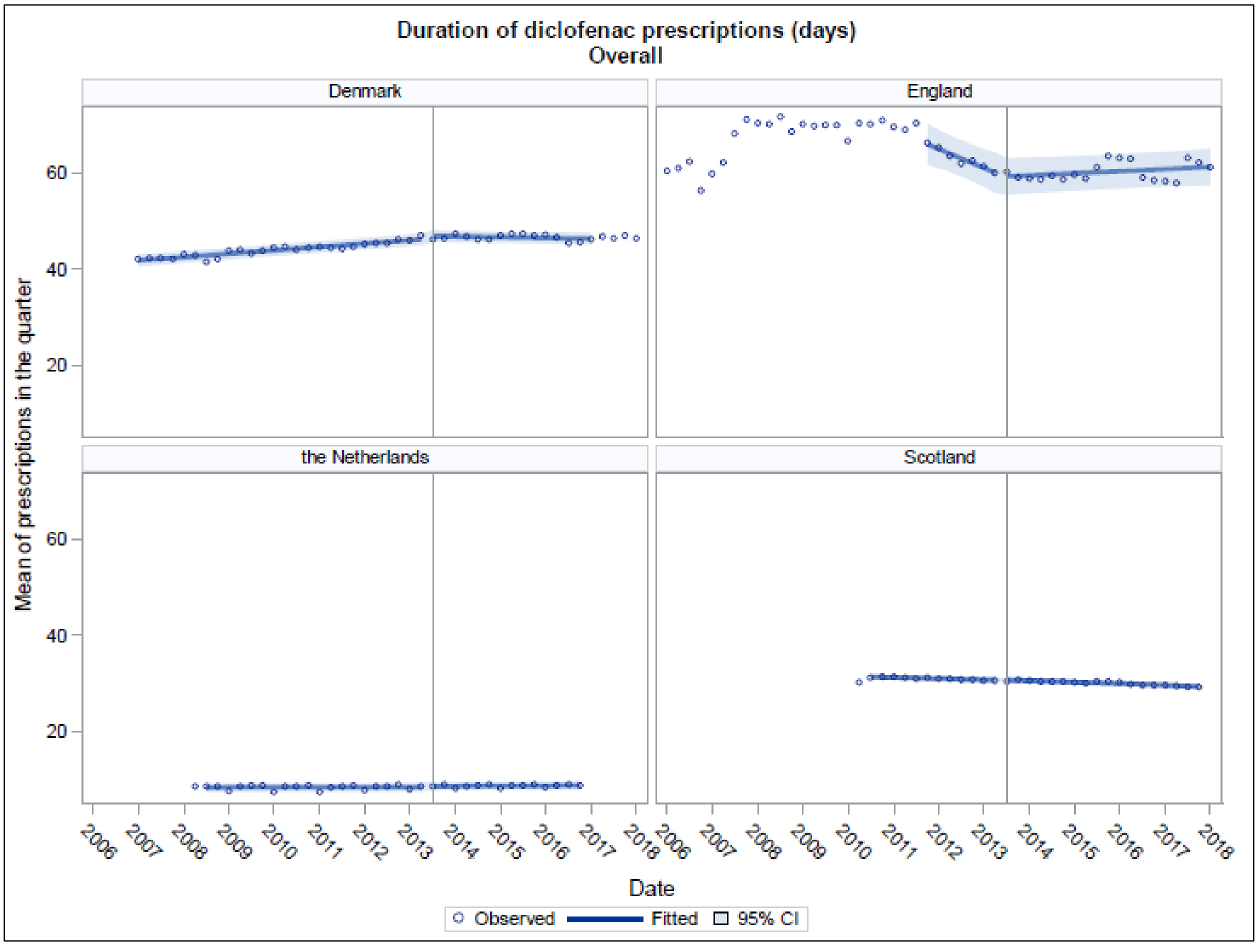

SUPPLEMENTARY TABLES

Table S1. Prevalence of diclofenac initiation at the beginning and end of follow-up for each country.

	**Prevalence (%)**		**Percentage change (%)**	
	**Baseline**	**Final**	**Absolute**	**Relative**
Initiation overall				
- Denmark	0.98	0.12	-0.86	-87.8
- England*	1.27	0.06	-1.21	-95.3
- Netherlands	2.47	1.73	-0.74	-30.0
- Scotland	1.18	0.31	-0.87	-73.7

Quarters: 2007Q1 to 2018Q1 in Denmark; 2008Q2 to 2016Q4 in The Netherlands; 2007Q1 to 2018Q1 in England; and 2010Q3 to 2017Q4 in Scotland. *~10% patients were from Northern Ireland and Wales

Table S2. Interrupted time series regression results for trends in diclofenac initiation in Denmark by indication, age, gender, exposure type and prescription duration.

	Trends in diclofenac initiation rates (%/quarter)		
	**Before June 2013**	**Change in first quarter after June 2013**	**Change after June 2013**
Indication			
- Crystal arthropathies	0.000 (-0.000, 0.000), p=0.374	-0.001 (-0.002, 0.000), p=0.173	-0.000 (-0.001, 0.000), p=0.152
- Inflammatory arthropathies	0.001 (-0.000, 0.001), p=0.107	-0.002 (-0.004, 0.000), p=0.081	-0.001 (-0.002,-0.000), p=0.036
- Osteoarthritis	0.003 (0.000, 0.005), p=0.031	-0.009 (-0.017,-0.002), p=0.020	-0.004 (-0.006,-0.001), p=0.008
Age			
- 0-17	-0.000 (-0.004, .004), p=0.843	0.003 (-0.009, 0.015), p=0.612	-0.001 (-0.005, 0.003), p=0.686
- 18-29	-0.003 (-0.022, 0.015), p=0.711	-0.006 (-0.060, 0.048), p=0.818	-0.003 (-0.022, 0.015), p=0.722
- 30-39	-0.001 (-0.024, 0.022), p=0.940	-0.023 (-0.090, 0.045), p=0.486	-0.009 (-0.032, 0.015), p=0.442
- 40-49	0.005 (-0.021, 0.030), p=0.692	-0.042 (-0.117, 0.034), p=0.257	-0.017 (-0.042, 0.009), p=0.188
- 50-59	0.014 (-0.018, 0.046), p=0.356	-0.075 (-0.168, 0.019), p=0.109	-0.025 (-0.057, 0.007), p=0.119
- 60-69	0.012 (-0.015, 0.039), p=0.353	-0.069 (-0.148, 0.010), p=0.081	-0.023 (-0.050, 0.004), p=0.089
- 70-79	0.026 (-0.005, 0.058), p=0.097	-0.116 (-0.209,-0.024), p=0.017	-0.039 (-0.071,-0.007), p=0.020
- 80+	0.008 (-0.018, 0.034), p=0.525	-0.063 (-0.140, 0.014), p=0.101	-0.017 (-0.043, 0.009), p=0.192
Gender			
- Female	0.005 (-0.019, 0.029), p=0.652	-0.040 (-0.110, 0.031), p=0.250	-0.014 (-0.038, 0.010), p=0.237
- Male	0.007 (-0.009, 0.023), p=0.391	-0.037 (-0.084, 0.011), p=0.119	-0.014 (-0.030, 0.002), p=0.086
Exposure type			
- One-off	0.004 (-0.012, 0.020), p=0.623	-0.025 (-0.073, 0.022), p=0.271	-0.010 (-0.026, 0.006), p=0.207
- Sporadic	Not available	Not available	Not available
- Chronic	0.002 (-0.001, 0.006), p=0.194	-0.012 (-0.023,-0.002), p=0.023	-0.004 (-0.007,-0.000), p=0.034
Prescription duration (days)	0.175 (0.145, 0.204), p=<.001	0.517 (-0.204, 1.238), p=0.155	-0.213 (-0.287,-0.139), p=<.001

Table S3. Interrupted time series regression results for trends in diclofenac initiation in the Netherlands by indication, age, gender, exposure type and prescription duration.

	Trends in diclofenac initiation rates (%/quarter)		
	**Before June 2013**	**Change in first quarter after June 2013**	**Change after June 2013**
Indication			
- Crystal arthropathies	0.003 (0.002, 0.003), p=<.001	-0.007 (-0.015, 0.000), p=0.063	-0.002 (-0.003,-0.001), p=<.001
- Inflammatory arthropathies	0.001 (0.000, 0.001), p=<.001	-0.003 (-0.006,-0.001), p=0.008	-0.001 (-0.001,-0.000), p=<.001
- Osteoarthritis	0.006 (0.005, 0.007), p=<.001	-0.025 (-0.038,-0.013), p=<.001	-0.005 (-0.006,-0.004), p=<.001
Age			
- 0-17	0.006 (0.002, 0.009), p=0.008	-0.079 (-0.141,-0.017), p=0.014	-0.008 (-0.016,-0.001), p=0.025
- 18-29	0.001 (-0.012, 0.013), p=0.877	-0.281 (-0.470,-0.093), p=0.005	-0.019 (-0.041, 0.003), p=0.087
- 30-39	-0.002 (-0.020, 0.016), p=0.823	-0.387 (-0.668,-0.106), p=0.009	-0.027 (-0.059, 0.006), p=0.109
- 40-49	0.002 (-0.019, 0.023), p=0.862	-0.475 (-0.805,-0.145), p=0.006	-0.039 (-0.078,-0.000), p=0.048
- 50-59	-0.000 (-0.024, 0.024), p=0.988	-0.587 (-0.952,-0.222), p=0.003	-0.040 (-0.082, 0.002), p=0.061
- 60-69	-0.006 (-0.030, 0.017), p=0.576	-0.609 (-0.951,-0.268), p=0.001	-0.036 (-0.075, 0.004), p=0.076
- 70-79	-0.012 (-0.035, 0.012), p=0.332	-0.645 (-0.992,-0.298), p=<.001	-0.039 (-0.079,-0.000), p=0.050
- 80+	-0.020 (-0.039,-0.002), p=0.035	-0.405 (-0.669,-0.142), p=0.004	-0.027 (-0.057, 0.003), p=0.080
Gender			
- Female	0.002 (-0.015, 0.018), p=0.826	-0.443 (-0.693,-0.194), p=0.001	-0.034 (-0.063,-0.005), p=0.024
- Male	-0.001 (-0.017, 0.014), p=0.864	-0.388 (-0.626,-0.149), p=0.002	-0.025 (-0.053, 0.003), p=0.078
Exposure type			
- One-off	0.001 (-0.011, 0.013), p=0.886	-0.320 (-0.507,-0.133), p=0.002	-0.023 (-0.045,-0.001), p=0.037
- Sporadic	-0.001 (-0.002, 0.001), p=0.421	-0.037 (-0.059,-0.014), p=0.002	0.002 (-0.001, 0.004), p=0.159
- Chronic	0.000 (-0.003, 0.003), p=0.993	-0.060 (-0.104,-0.016), p=0.010	-0.008 (-0.013,-0.003), p=0.003
Prescription duration (days)	0.005 (-0.027, 0.037), p=0.750	0.149 (-0.419, 0.717), p=0.597	0.005 (-0.058, 0.069), p=0.861

Table S4. Interrupted time series regression results for trends in diclofenac initiation in the England by indication, age, gender, exposure type and prescription duration.

	Trends in diclofenac initiation rates (%/quarter)		
	Before June 2013	Change in first quarter after June 2013	Change after June 2013
Indication			
- Crystal arthropathies	0.000 (-0.000, 0.000), p=0.527	-0.001 (-0.001,-0.000), p=<.001	-0.000 (-0.000, 0.000), p=0.143
- Inflammatory arthropathies	-0.000 (-0.000,-0.000), p=<.001	-0.001 (-0.002,-0.001), p=<.001	0.000 (0.000, 0.000), p=0.032
- Osteoarthritis	-0.002 (-0.003,-0.002), p=<.001	-0.017 (-0.022,-0.013), p=<.001	0.002 (0.001, 0.002), p=0.002
Age			
- 0-17	-0.002 (-0.003,-0.001), p=0.002	-0.005 (-0.011, 0.000), p=0.056	0.001 (0.000, 0.002), p=0.028
- 18-29	-0.018 (-0.021,-0.015), p=<.001	-0.061 (-0.076,-0.046), p=<.001	0.013 (0.010, 0.016), p=<.001
- 30-39	-0.028 (-0.032,-0.024), p=<.001	-0.101 (-0.121,-0.081), p=<.001	0.020 (0.016, 0.024), p=<.001
- 40-49	-0.028 (-0.032,-0.023), p=<.001	-0.154 (-0.178,-0.131), p=<.001	0.018 (0.013, 0.023), p=<.001
- 50-59	-0.029 (-0.035,-0.023), p=<.001	-0.152 (-0.183,-0.121), p=<.001	0.019 (0.013, 0.026), p=<.001
- 60-69	-0.025 (-0.031,-0.018), p=<.001	-0.125 (-0.158,-0.092), p=<.001	0.017 (0.010, 0.023), p=<.001
- 70-79	-0.013 (-0.021,-0.005), p=0.003	-0.114 (-0.155,-0.074), p=<.001	0.007 (-0.001, 0.016), p=0.074
- 80+	-0.007 (-0.011,-0.004), p=<.001	-0.050 (-0.068,-0.032), p=<.001	0.005 (0.001, 0.008), p=0.012
Gender			
- Female	-0.020 (-0.023,-0.017), p=<.001	-0.101 (-0.118,-0.085), p=<.001	0.014 (0.010, 0.017), p=<.001
- Male	-0.018 (-0.021,-0.014), p=<.001	-0.085 (-0.103,-0.067), p=<.001	0.011 (0.008, 0.015), p=<.001
Exposure type			
- One-off	-0.014 (-0.016,-0.012), p=<.001	-0.073 (-0.085,-0.060), p=<.001	0.009 (0.007, 0.012), p=<.001
- Sporadic	-0.000 (-0.000, 0.000), p=0.413	0.000 (-0.000, 0.000), p=0.151	0.000 (-0.000, 0.000), p=0.619
- Chronic	-0.005 (-0.006,-0.003), p=<.001	-0.021 (-0.027,-0.014), p=<.001	0.003 (0.002, 0.004), p=<.001
Prescription duration (days)	-0.983 (-1.640,-0.327), p=0.005	0.199 (-3.111, 3.510), p=0.902	1.092 (0.420, 1.764), p=0.003

~10% patients were from Northern Ireland and Wales

Table S5. Interrupted time series regression results for trends in diclofenac initiation in the Scotland by indication, age, gender, exposure type and prescription duration.

	Trends in diclofenac initiation rates (%/quarter)		
	**Before June 2013**	**Change in first quarter after June 2013**	**Change after June 2013**
Indication			
- Crystal arthropathies	0.000 (0.000, 0.000), p=<.001	-0.001 (-0.001,-0.001), p=<.001	-0.000 (-0.000,-0.000), p=<.001
- Inflammatory arthropathies	0.000 (0.000, 0.000), p=<.001	-0.002 (-0.003,-0.002), p=<.001	-0.000 (-0.000,-0.000), p=<.001
- Osteoarthritis	0.001 (0.001, 0.001), p=<.001	-0.015 (-0.017,-0.013), p=<.001	-0.001 (-0.002,-0.001), p=<.001
Age			
- 0-17	0.002 (0.000, 0.004), p=0.041	-0.048 (-0.065,-0.030), p=<.001	-0.005 (-0.007,-0.002), p=<.001
- 18-29	0.020 (0.005, 0.034), p=0.009	-0.567 (-0.690,-0.445), p=<.001	-0.051 (-0.067,-0.035), p=<.001
- 30-39	0.014 (-0.006, 0.033), p=0.164	-0.799 (-0.967,-0.632), p=<.001	-0.058 (-0.080,-0.036), p=<.001
- 40-49	0.019 (-0.004, 0.041), p=0.097	-1.031 (-1.226,-0.836), p=<.001	-0.071 (-0.096,-0.046), p=<.001
- 50-59	0.008 (-0.015, 0.030), p=0.487	-1.083 (-1.278,-0.888), p=<.001	-0.053 (-0.078,-0.028), p=<.001
- 60-69	-0.012 (-0.027, 0.003), p=0.122	-0.838 (-0.969,-0.706), p=<.001	-0.018 (-0.034,-0.001), p=0.039
- 70-79	-0.014 (-0.025,-0.003), p=0.015	-0.567 (-0.665,-0.469), p=<.001	-0.002 (-0.014, 0.010), p=0.750
- 80+	-0.010 (-0.016,-0.005), p=<.001	-0.272 (-0.320,-0.223), p=<.001	0.004 (-0.002, 0.010), p=0.221
Gender			
- Female	0.005 (-0.009, 0.018), p=0.504	-0.706 (-0.827,-0.585), p=<.001	-0.037 (-0.053,-0.022), p=<.001
- Male	0.013 (-0.001, 0.027), p=0.070	-0.633 (-0.753,-0.513), p=<.001	-0.041 (-0.056,-0.025), p=<.001
Exposure type			
- One-off	0.011 (0.001, 0.021), p=0.039	-0.518 (-0.608,-0.429), p=<.001	-0.035 (-0.046,-0.023), p=<.001
- Sporadic	-0.000 (-0.000, 0.000), p=0.204	-0.008 (-0.010,-0.006), p=<.001	-0.000 (-0.001, 0.000), p=0.062
- Chronic	-0.003 (-0.006, 0.001), p=0.115	-0.145 (-0.175,-0.115), p=<.001	-0.003 (-0.007, 0.001), p=0.089
Prescription duration (days)	-0.063 (-0.090,-0.035), p=<.001	0.095 (-0.160, 0.350), p=0.449	-0.015 (-0.047, 0.016), p=0.326

Table S6. List of medicines used in the study.

ATC code	Drug names
**Other systemic Non-steroidal anti-inflammatory drugs**	
M01AB16	ACECLOFENAC
M01AB11	ACEMETACIN
N02BA01	ASPIRIN
M01AH01	CELECOXIB
M01AE14	DEXIBUPROFEN
M01AE17	DEXKETOPROFEN
M01AB05	DICLOFENAC POTASSIUM
M01AB05	DICLOFENAC SODIUM
M01AB08	ETODOLAC
M01AH05	ETORICOXIB
M01AE04	FENOPROFEN
M01AE09	FLURBIPROFEN
M01AE01	IBUPROFEN
M01AB01	INDOMETACIN
M01AE03	KETOPROFEN
M01AB15	KETOROLAC TROMETAMOL
M01AG01	MEFENAMIC ACID
M01AC06	MELOXICAM
M01AX01	NABUMETONE
M01AE02	NAPROXEN
M01AH04	PARECOXIB
M01AC01	PIROXICAM
M01AB02	SULINDAC
M01AC02	TENOXICAM
M01AE11	TIAPROFENIC ACID
**Topical NSAIDs**	
M02AA15	DICLOFENAC
M02AA08	FELBINAC
M02AA13	IBUPROFEN
M02AA10	KETOPROFEN
M02AA07	PIROXICAM
**Paracetamol**	
N02BE01	paracetamol
**Opioids (licensed for or likely to be used in chronic pain)**	
N02AE01	BUPRENORPHINE
R05DA04	CODEINE PHOSPHATE

N07BC06	DIAMORPHINE HYDROCHLORIDE (subcutaneous)
N02AA08	DIHYDROCODEINE TARTRATE
N02AB03	FENTANYL (transdermal)
N02AX05	MEPTAZINOL
N02AA01	MORPHINE rectal / parenteral
N02AA01	MORPHINE oral
N02AA05	OXYCODONE HYDROCHLORIDE oral
N02AA05	OXYCODONE HYDROCHLORIDE parenteral
N02AD01	PENTAZOCINE
N02AB02	PETHIDINE HYDROCHLORIDE
N02AX06	TAPENTADOL
N02AX02	TRAMADOL HYDROCHLORIDE
**Other chronic pain medication**	
N02BG06	NEFOPAM HYDROCHLORIDE
N06AA09	AMITRIPTYLINE HYDROCHLORIDE
N03AX16	PREGABALIN
N06AA10	NORTRIPTYLINE
N03AX12	GABAPENTIN
N01BB02	LIDOCAINE HYDROCHLORIDE (medicated plasters)
M02AB01	CAPSAICIN
